# Association between Predicted Effects of *TP53* Missense Variants on Protein Conformation and Their Phenotypic Presentation as Li-Fraumeni Syndrome or Hereditary Breast Cancer

**DOI:** 10.3390/ijms22126345

**Published:** 2021-06-14

**Authors:** Yaxuan Liu, Olga Axell, Tom van Leeuwen, Robert Konrat, Pedram Kharaziha, Catharina Larsson, Anthony P. H. Wright, Svetlana Bajalica-Lagercrantz

**Affiliations:** 1 Department of Oncology-Pathology, Karolinska Institutet, Bioclinicum, Karolinska University Hospital, 17164 Stockholm, Sweden; olga.axell@outlook.com (O.A.); kharaziha@gmail.com (P.K.); Catharina.Larsson@ki.se (C.L.); svetlana.lagercrantz@ki.se (S.B.-L.); 2 Department of Laboratory Medicine, Division of Biomolecular and Cellular Medicine, Karolinska Institutet, 17177 Stockholm, Sweden; tom.van.leeuwen@stud.ki.se (T.v.L.); anthony.wright@ki.se (A.P.H.W.); 3 Christian Doppler Laboratory for High-Content Structural Biology and Biotechnology, Department of Structural and Computational Biology, Max F. Perutz Laboratories, University of Vienna, 1030 Vienna, Austria; robert.konrat@univie.ac.at

**Keywords:** Li-Fraumeni syndrome, hereditary breast cancer, germline *TP53* missense variants, quantitative prediction model, protein conformation

## Abstract

Rare germline pathogenic *TP53* missense variants often predispose to a wide spectrum of tumors characterized by Li-Fraumeni syndrome (LFS) but a subset of variants is also seen in families with exclusively hereditary breast cancer (HBC) outcomes. We have developed a logistic regression model with the aim of predicting LFS and HBC outcomes, based on the predicted effects of individual *TP53* variants on aspects of protein conformation. A total of 48 missense variants either unique for LFS (*n* = 24) or exclusively reported in HBC (*n* = 24) were included. LFS-variants were over-represented in residues tending to be buried in the core of the tertiary structure of TP53 (*p* = 0.0014). The favored logistic regression model describes disease outcome in terms of explanatory variables related to the surface or buried status of residues as well as their propensity to contribute to protein compactness or protein-protein interactions. Reduced, internally validated models discriminated well between LFS and HBC (C-statistic = 0.78−0.84; equivalent to the area under the ROC (receiver operating characteristic) curve), had a low risk for over-fitting and were well calibrated in relation to the known outcome risk. In conclusion, this study presents a phenotypic prediction model of LFS and HBC risk for germline *TP53* missense variants, in an attempt to provide a complementary tool for future decision making and clinical handling.

## 1. Introduction

Li-Fraumeni syndrome (LFS) is a rare heritable extreme tumor risk syndrome characterized mainly by premenopausal breast cancer, soft tissue sarcoma, brain tumors, osteosarcoma and adrenocortical carcinoma, and was first described in 1969 [1]. LFS was subsequently shown to be associated with a germline *TP53* variant [2]. As more families with a variety of tumors were reported, less restricted criteria became used to define Li-Fraumeni-like (LFL) families [3] that did not meet the classical LFS criteria but were suggestive of LFS, with a detection rate for germline *TP53* alterations of 20–40% in LFL as compared to 70% in LFS [4]. At present, the most commonly used screening criteria are the Chompret criteria, with a detection rate of 29%, since they include a large group of patients for screening [5]. For example, according to these criteria a patient with breast cancer below 31 years, should be screened irrespective of family history. With the increased use of cancer gene panels in genetic testing, the detection of pathogenic *TP53* variants has increased, and up to 1% of families with exclusively hereditary breast cancer (HBC) have been shown to carry a germline *TP53* variant [6].

The wide range of phenotypic presentation associated with germline *TP53* variants makes tumor risk assessment difficult and genetic counseling challenging in these patients and families. Moreover, 7–20% of constitutional *TP53* alterations are *de novo* [7], and thus presented in individuals without a family history of the disease. Due to the lack of knowledge about predicting genotype-phenotype association, all germline carriers are recommended a thorough surveillance program including yearly whole-body magnetic resonance imaging (MRI) examinations with the result that a large proportion of *TP53* variant carriers are exposed to unnecessary examinations [8,9].

The TP53 protein is a transcription factor that binds as a tetramer to DNA, and activates a large number of genes that promote DNA repair mechanisms or apoptosis including cell cycle regulatory proteins and members of the Bcl-2 family [10,11]. Each monomer is divided into different structural and functional domains, including a transactivation domain, a proline-rich region, a DNA binding domain (DBD), a oligomerization domain, a nuclear localization signal and a C-terminal regulatory domain [12]. TP53 plays a critical role in genomic homeostasis, and its activities are tightly regulated by a network of protein-protein interactions, microRNAs, and a range of post-translational modifications, including phosphorylation, acetylation, methylation and ubiquitination [13]. 

About two thirds of reported germline *TP53* variants are single site missense changes, predominantly located in the DBD [14]. Carriers are heterozygous for the *TP53* variant thus possessing both wild-type and variant monomers allowing formation of hetero-tetramers that result in a dominant-negative functional effect of some variants [15]. It has been suggested that patients with missense variants have earlier age of tumor onset (23.8 years), compared to those with loss of function variants (28.5 years) [16]. Moreover, unequal penetrance of missense variants is known in LFS where, for example, 58% of carriers with R248W (amino acid change at residue 248 from arginine to tryptophan) compared to only 21% of carriers with R231Q develop tumors before 30 years of age [17]. 

The TP53 DBD consists of a beta-sandwich tertiary structure with two antiparallel beta-sheets, that serve to orientate and stabilize the loop-sheet-helix DNA-binding motif [18]. Contacts with DNA are mainly to the sugar-phosphate backbone of the DNA helix (K120, S241, R248, R273, A276, R283) as well as a smaller number of contacts to specific bases within the consensus pentamer binding sequence (C277, R280 and K120). Other residues that are also mutated in sporadic tumors are important for anchoring the DNA binding motif to the beta-sandwich structure (e.g., R175, G245, R249, R282) or for stabilizing the beta-sandwich structure (e.g., V143, V157, Y220, F270). Although the TP53 DBD folds into a compact tertiary structure at body temperature, it is thermally unstable and unfolds at only slightly higher temperatures (>40 °C) or in response to tumor associated *TP53* variants [19,20]. Interestingly, some novel pharmaceutical agents (e.g., CP-31398 and APR-246) have been shown to restore wild-type functionality to mutant TP53 proteins by increasing their thermal stability [21]. Different missense variants of the DBD have different effects on protein conformation and its mechanistic characteristics and, interestingly, for some sporadic tumors a relationship between the effect of variants on mechanistic aspects of TP53 function and the type of tumor has been observed (e.g., glioblastoma vs. adrenocortical carcinoma) [22]. It is therefore possible that differential effects of germline *TP53* variants on conformational aspects of TP53 and its functionality could contribute to differences in phenotypes (e.g., LFS vs. HBC).

In this study we investigate whether the phenotypic outcome observed for different *TP53* variants can be accounted for by the differential effects of the variants on TP53 protein conformation as well as whether variant associated protein conformation changes can be used to predict disease outcome.

## 2. Materials and Methods

### 2.1. Selection of TP53 Variants

Included variants and their clinical characteristics were selected from publicly available databases and publications as described below. All *TP53* variants that were defined as LFS in our cohort were identified through the IARC database, and were not found to be reported in a HBC-family there or elsewhere. According to the IARC database the families thus fulfilled the classic LFS criteria [23] upon screening. For the HBC cohort, 17 variants were identified through the IARC database, 17 from the meta-analysis by Fortuno et al. [24] and 2 from Kharaziha et al. [25]. However, 12 of the HBC variants were reported both in the IARC database and in Fortuno et al., thus resulting in 24 unique HBC variants.

The selection process for the *TP53* missense variants used in this study is summarized in Figure S1. A total of 24 germline *TP53* variants unique for LFS were selected by evaluation of 408 *TP53* variants in the IARC database (R20, January 2020) [26]. Out of 296 missense variants, 62 variants were LFS-class, while 58 were LFL-class and 78 variants were TP53-Chompret-class, according to the terminology used in the IARC database. 117 variants were present in the FH-class (family history of cancer which does not fulfill LFS or any of the LFL definitions), noFH-class (no family history of cancer) or the other-class (variants that were not included in other classes). Many variants were present in more than one class. We selected the variants uniquely classified as LFS (*n* = 24) to represent the LFS-variants used in the study and the amino acids involved are referred to as LFS-residues (Table S1). 

The non-redundant unified group of 24 HBC-specific germline *TP53* variants was selected from the IARC database, from the Fortuno et al. [24] meta-analysis of *TP53*-related HBC without a history of LFS and from the Kharaziha et al. [25] Swedish germline *TP53* cohort. Since there was no specified HBC-class in the IARC database, we selected the HBC-variants from FH-class, noFH-class and other-class and excluded those that overlapped with the LFS-class, LFL-class and TP53-Chompret-class. Further, the selected HBC *TP53* missense variants were exclusively reported in breast cancer. Out of 73 identified germline variants in Fortuno et al. [24], 41 were missense variants, of which 17 were also not present in the LFS-class, LFL-class or TP53-Chompret-class in the IARC database. In the Swedish cohort, reported by Kharaziha et al. [25], 24 germline *TP53* variants were identified, of which 6 missense variants were specifically found in HBC including two that were not present in the LFS-class, LFL-class or TP53-Chompret-class in the IARC database. The 24 resulting *TP53* variants were included in the study and the amino acids involved are referred to as HBC-residues (Table S1).

### 2.2. Analysis of Protein Structure

A published X-ray crystal structure of a tetrameric TP53 derivative containing the DBD fused to the oligomerization domain bound to the natural p21 TP53-response element (PDB accession number 3TS8) was used [27]. Details of the p53CR2 derivative used for crystallography have been fully described [28]. P53CR2 contains protein regions equivalent to residues 94–292 and 324–355 of TP53 and includes some stabilizing amino acid substitutions that distinguish the protein from the equivalent wild-type TP53 residues. PyMOL software (Schrödinger, https://pymol.org, 23 April 2019) was used to display HBC- and LFS-residues in the context of the tertiary structure of the protein and to identify different residue classes, using customized scripts adapted from those available in the PyMOL script library [29]. The findSurfaceResidues script [30] was adapted to allow identification of Buried (non-Surface) residues, defined as residues with a Solvent-Accessible Surface Area (SASA) below user-defined cutoff values. A cutoff value of 11Å^2^ defined approximately 30% of residues as Buried and was used to classify residues for statistical analysis. The interfaceResidues script [31] was adapted to allow identification of residues at the interface between TP53 monomers in the tetrameric structure or between TP53 monomers and DNA. Interactions are defined as regions where the overlapping Surface area between atoms from different molecules exceeds a cutoff area. The default cutoff value of 1.0Å^2^ was used. The ss script [32] was adapted to list residues in different types of secondary structure, defined as alpha-helix, beta-sheet or loop.

### 2.3. Explanatory Variables

Explanatory variables were a priori restricted to variables reflecting the effects of missense variants on different aspects of TP53 protein conformation (Table 1). Bur is a categorical variable defining Buried and Surface residues (as described above). A third Bur category (unknown) describes residues that are not included in the TP53 tertiary structure used. The remaining explanatory variables are continuous and are calculated using prediction algorithms for different aspects of protein conformation, which generate residue-by-residue scores for wild-type TP53 and each of the included TP53 variants. The value of these variables is defined as the difference between the variant and wild-type scores at the position of the substituted residue (variant score minus wild-type score). The prediction algorithms predict values that reflect propensity for intrinsic protein disorder, peptide-backbone flexibility, secondary structure, protein tertiary structure/compactness and protein interaction (Table 1). Protein interaction site prediction in the TP53 sequence was performed using a meta-structure-based homology method [33] as already reported [34] and summarized in the Supplementary Materials and methods. The analysis results in a residue specific score that is proportional to the propensity of a given residue to be part of a protein interaction site. The PPI6_dif variable used in regression models was calculated using more sensitive settings for predicting protein-interaction regions (minimum query segment = 6 amino acids), while less sensitive settings (minimum query segment = 10 amino acids) were used to identify and plot the most prominent TP53 protein-interaction regions (see Supplementary Materials and methods for details). 

The findSurfaceResidues script from Pymol software, version 2.3.1 [28]. was adapted to allow identification of Buried residues (<cutoff at 11Å^2^). Bur is a categorical variable including Buried, Surface (>cutoff at 11Å^2^) and unknown (not included in the TP53 tertiary structure used). The Espritz predictor [35] was trained using proteins in the Disprot database (disprot) as well as tertiary structures determined by nuclear magnetic resonance (nmr) or X-ray diffraction (xray). The IUPred2A [36] algorithm was run with the long disorder (iupl), short disorder (iups), structured domain (iupstr) and anchor (anc) arguments. Dynamine [37] predicts protein backbone flexibility (dyn). Meta-structure analysis [33,34] predicts values for two parameters, compactness (comp) and secondary structure (Sec). PPI6 uses Meta-structure values to predict residues in regions with propensity for protein-protein interactions.

The _dif suffix indicates that the variable is the difference between the value for variant TP53 and wild-type TP53 at the position of the substituted residue (variant score minus wild type score).

Abbreviations: DB, database; NMR, nuclear magnetic resonance; X-ray, Xray diffraction.

### 2.4. Statistical Analysis

Data were collected and processed using R software, version 3.6. Fisher’s exact test was used to evaluate whether LFS- or HBC-residues were over- or under-represented in residue sets reflecting different structural aspects of the TP53 protein structure. *p*-values were for two-sided tests and were adjusted for multiple testing, where appropriate, using the false discovery rate method. Associations between disease outcome (LFS or HBC) and the explanatory variables for different *TP53* variants as well as their predictive potential were evaluated by logistic regression using internal 1000-fold bootstrapped validation and a backwards step-down approach to variable number reduction as implemented in the rms-package (validate function: method = “boot”, B = 1000, bw = TRUE, rule = “p”, type = “individual”, sls = 0.13). The validate function delivers values for a number of parameters relevant for assessing the discrimination performance of models and the risk for overfitting, including the concordance statistic (C-statistic). Similarly, the calibrate function (method = “boot”, B = 1000) was used to test the quality of model calibration. Further validation was performed using leave-one-out cross validation by using the validate function (arguments as above except sls = 0.16) to produce reduced models for each combination of n-1 variants. Predictions were expressed as probability of LFS (predict function, type = ”fitted”). ROC curves were produced using the ROCit package. The favored model was described visually using a nomogram and its potential utility was evaluated by decision curve analysis (rmda package) [38]. *p* < 0.05 was considered as the threshold for statistical significance unless stated otherwise. 

## 3. Results

### 3.1. Characteristics of LFS and HBC Germline TP53 Variants

A total of 48 germline *TP53* missense variants were selected from the IARC database, Fortuno et al. [24] and Kharaziha et al. [25] including 24 uniquely observed in LFS and 24 exclusively reported in HBC (Figure S1). The source, number of patients and families as well as the type of LFS-core tumor types obsrved for each of the 48 variants are detailed in Table S1. 

**Figure 1:**
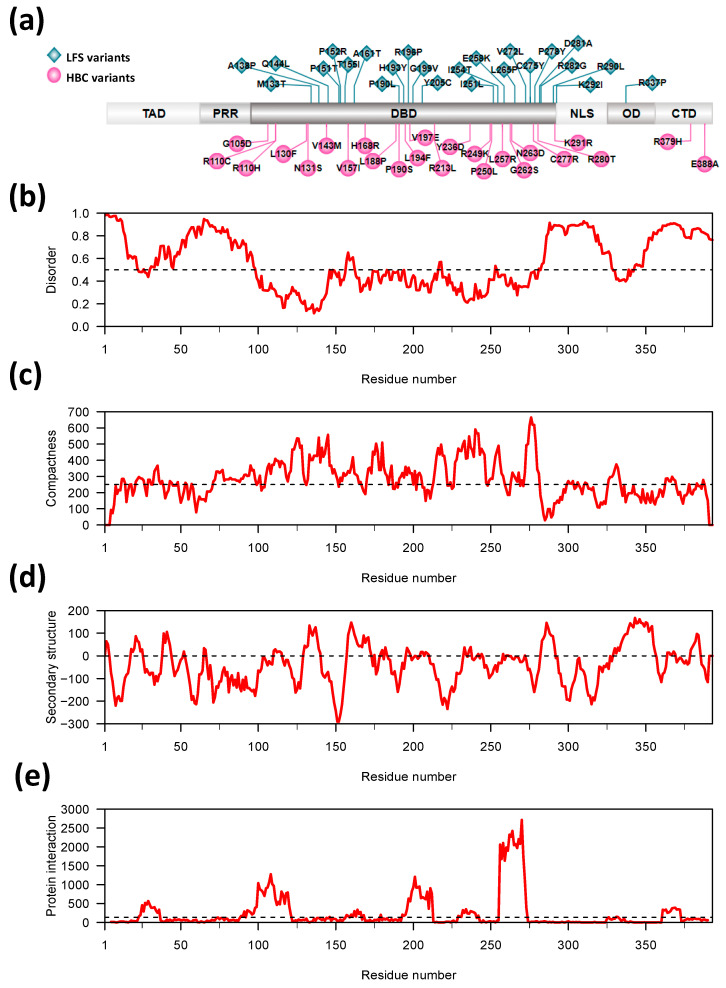
Location of *TP53* missense variants in the TP53 protein sequence and in relation to its predicted disorder. (**a**). Schematic illustration of the TP53 amino acid sequence and protein domains with the location of the 24 LFS variants shown above (cyan green-blue rhombus) and the 24 HBC-variants indicated below (magenta purple-red circles) [39]. The TP53 domains are illustrated for the transactivation domain (TAD), the proline-rich region (PRR), the DNA binding domain (DBD), the nuclear localization signal (NLS), the oligomerization domain (OD) and the C-terminal regulatory domain (CTD). (**b**) Predicted disorder profile of wild type TP53. The IUPred2A predictor was used with the “long” argument. Scores > 0.5 (above dotted line) indicate disordered regions. The approximate location of the DBD and OD are shown (grey shading). (**c**) Predicted compactness of wild type TP53. The dotted line at a value of 250 (*y*-axis) emphasizes the higher compactness values predicted for the DBD. The approximate location of the DBD and OD are shown (grey shading). (**d**) Predicted secondary structure of wild type TP53. Values > 0 (dotted line) are predicted to be alpha-helical and values < 0 are predicted to have beta-strand conformation. The approximate location of the DBD and OD are shown (grey shading). (**e**) Predicted regions with protein interaction propensity in wild type TP53. The dotted line shows a level equivalent to 5% of the maximum value. Apparently artefactual values for the first 4 residues and last 3 residues of TP53 were omitted. The approximate location of the DBD and OD are shown (grey shading).

The vast majority of variants were mapped to the DBD of the TP53 protein (Figure 1a). Specifically, 23 variants in the LFS-group were located in the DBD and one in the oligomerization domain. In the HBC-group 22 variants were in the DBD, and two in the C-terminal regulatory domain. Figure 1b shows that most of the LFS- and HBC-residues are located in regions predicted to have an ordered protein conformation, however, the prediction values are generally close to the threshold of 0.5 for transition to predicted conformational disorder. This is consistent with previous reports showing low conformational stability of the DNA-binding domain tertiary structure [12,20]. Consistently, the meta-structure prediction method [33] predicts a higher degree of compactness in the DBD (Figure 1c) and correctly predicts a predominance of beta-sheet conformation in the DBD as well as the alpha-helical nature of the oligomerization domain (Figure 1d). Finally, the three most predominant predicted protein interaction domains are in the DBD and the two most C-terminal of these coincide with regions containing clusters of variant LFS- and HBC-residues (Figure 1e).

### 3.2. Location of LFS- and HBC-Residues in Relation to the TP53 Protein Structure

**Figure 2:**
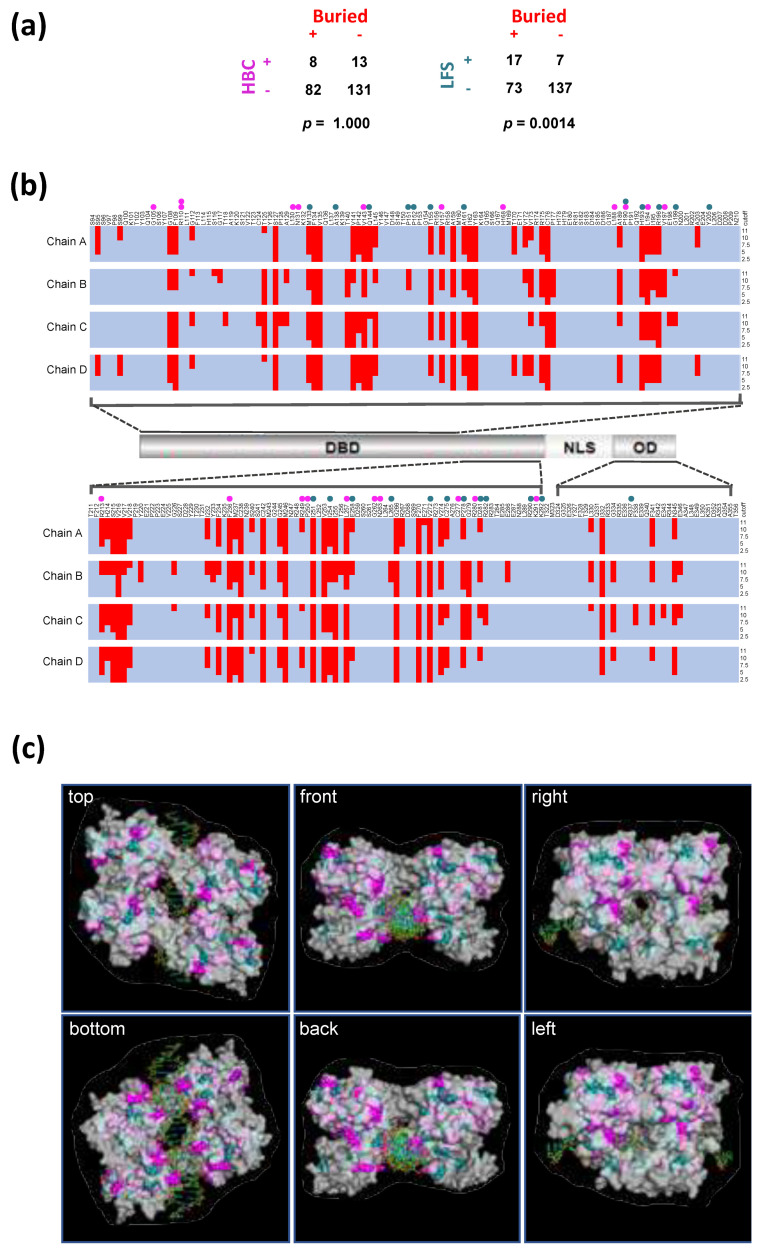
Comparison of surface exposure of *TP53* missense variants in LFS and HBC. (**a**) LFS-residues are significantly enriched in Buried residues with lower surface exposure (<cutoff at 11Å^2^) than expected by chance while HBC-residues are not. Contingency tables and respective *p*-values are shown (Fisher’s Exact Test, two-sided). Residues were defined as Buried if their surface area was below the cutoff value in any of the 4 TP53 monomers in the TP53 structure (PDB name = 3TS8). The HBC-residues R379 and E388 are not included in this TP53 structure, and the R110 was calculated once, therefore only 21 HBC-residues were included. (**b**) Location of Buried residues (red shading) in a TP53 derivative containing the DBD and OD that was used for tertiary structure determination (3TS8). Cutoff values (Å^2^) to distinguish between Surface (>cutoff) and Buried residues (<cutoff) were 11 (used in the statistical test in (**a**), 10, 7.5, 5 and 2.5 (see right side of panel). Results are shown for each of the monomers (Chains A–D) within the TP53 tetramer bound to DNA. LFS-residues and HBC-residues are indicated by cyan and magenta filled circles respectively. (**c**) Localization of LFS-residues (cyan) and HBC-residues (magenta) in relation to the surface of the TP53 tetramer bound to DNA (3TS8, transparent grey). Stronger color indicates Surface exposure while weaker color indicates parts of residues that are below the protein surface (Buried). The 6 panels show all views of the protein caused by stepwise 90° rotations of the “top” structure.

Variant LFS- and HBC-residues are distributed and juxtaposed throughout the DBD located in the central part of TP53 with no apparent pattern associated with either disease outcome. We considered whether there might be associations with secondary-structure elements (alpha-helix, beta-sheet or disordered regions), tertiary structure aspects (such as Surface or Buried locations) or quaternary structure aspects (DNA interacting residues or inter-monomer protein interacting residues in the context of the TP53 tetramer). For each of the TP53 monomers (Chain A, B, C and D), there was a significant enrichment of variant LFS-residues in the approximately 30% of residues that are Buried (i.e., least Surface exposed) in the crystal structure of the wild-type TP53 tetramer bound to DNA (Table S2). A similar result was also obtained when the Buried (non-Surface) classification of residues was combined for all four monomers (Figure 2a). No other significant associations were found (Table S2).

Figure 2b shows the location of LFS- and HBC-residues in relation to the location of Buried residues defined by different cutoff values used to define the threshold Surface-exposed area per residue. As summarized in Figure 2a, there is a clear tendency for LFS-residues to be Buried, while no such association was found for HBC-residues. The same pattern is seen in Figure 2c, which shows LFS- and HBC-residues in the context of the tertiary structure of the TP53 tetramer bound to DNA.

### 3.3. Association between TP53-Variant-Induced Changes in Protein Conformation Characteristics and Disease Outcome

The enrichment of LFS-residues in the set of most Buried TP53 residues suggests that the disease associated variants might tend to alter the folded conformation of TP53 in LFS patients. The more even distribution of HBC-residues on the Surface and core of the protein structure would give a greater possibility for disease associated variants to disrupt or modify interactions between TP53 and its DNA or protein ligands in HBC patients. To investigate these aspects further we made multivariate models to predict disease outcome as a function of Buried vs. Surface status for LFS- and HBC-residues, together with a range of variables predicting the effect of the variant residues on protein conformation aspects, such as intrinsic disorder, protein backbone flexibility, propensity for tertiary structure formation, propensity for secondary structure formation and protein interaction propensity (Table 1). Several of these protein conformation aspects appear to be of potential relevance (Figure 1b–d) and values for all variables are listed in Table S3 and Table S3 Appendix.

**Figure 3:**
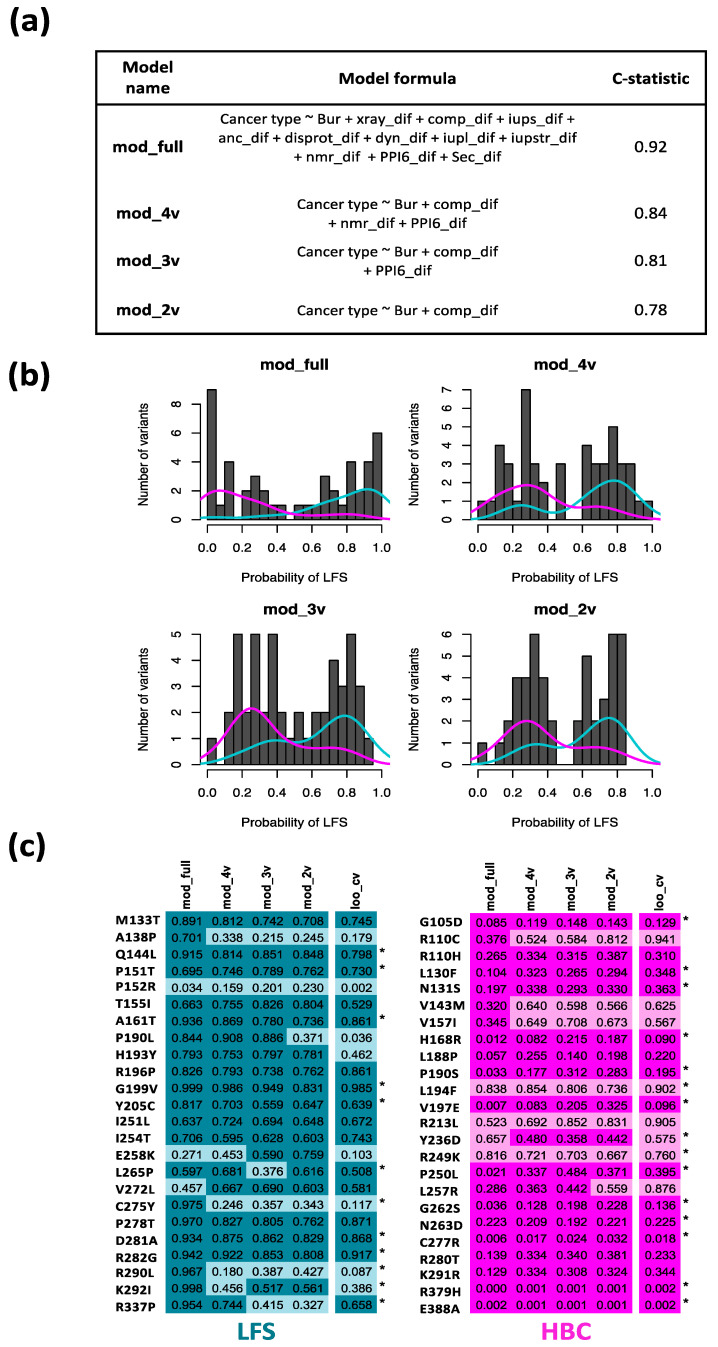
Protein conformation parameters are associated with disease phenotype and may have predictive value. (**a**) Multivariate logistic regression models for prediction of phenotype class (LFS or HBC) using a range of available protein conformation related explanatory variables describing different protein conformation aspects (full model, mod_full). A reduced model (mod_4v) was produced by stepwise variable exclusion from the full model (rms package). Further reduction was done by progressive manual removal of the least well performing variable to produce models with 3 and 2 explanatory variables, respectively (mod_3v and mod_2v). Indicators of model performance (C-statistic) are shown. (**b**) Bimodal probability distributions for models, showing the overall separation of output variables (LFS and HBC). Histograms show the distribution of the predicted probabilities of residues causing LFS, for the different models. The overall separation of variants as LFS (cyan line) or HBC (magenta line) are shown for each model. (**c**) Probability values for individual LFS (cyan) and HBC (magenta) variants produced by the full model and reduced models (columns 1–4 in each panel) as well as leave-one-out cross validation results (loo_cv) in which each respective variant is left out from a reduced model that is then used to predict the outcome associated with the left-out variable (column 5 in each panel). An asterisk (*) indicates that the loo_cv model contains the same variables as the mod_4v model (model details and results for each of the loo_cv model are tabulated in Table S5). Based on the binomial distribution minima in part b, probability values >0.5 for LFS and <0.5 for HBC are colored darker to give an indication of the relative performance (correct predictions) of the different models as well as how performance is affected in the cross-validation procedure in which predictions are made for each individual variant by models excluding data for the predicted variant. Variant/model combinations with lighter color indicate incorrect predictions.

Since the disease outcome is defined by a binary variable (LFS or HBC) we used a logistic regression approach. The relatively small number of variants for each outcome (n = 24 in each group) imposed limitations on the number of explanatory variables (*n* = 2 to 4) that could reasonably be included in a final model. First a full model (mod_full), likely associated with overfitting problems, was made using 12 explanatory variables describing the effects of the LFS- and HBC-variants on different aspects of protein conformation. An internal bootstrap cross-validation procedure was then used to produce a reduced model by removing less useful variables in a stepwise manner. The reduced model produced during the cross-validation procedure contained 4 variables (mod_4v). Further variable number reduction was done manually by successively removing the variable with the least significant beta coefficient to produce models with three and two explanatory variables, mod_3v and mod_2v, respectively (Figure 3a, Table S4). The performance of the models, as shown by the C-statistic, is lower in the reduced models than the full model as expected, but they are still in the vicinity of the level required for a useful predictive model. The corresponding ROC curves are shown in Figure S2.

Figure 3b shows the binomial distribution of prediction probabilities for the models with all showing a minima close to a probability of 0.5. The figure also shows the relative distribution of the known disease outcomes in relation to the probability distributions. Even though the full model shows a higher degree of discrimination between the LFS and HBC outcomes than the reduced models, the full model is likely associated with overfitting issues. The reduced models none-the-less show correct prediction of most variants with a much lower risk of potential for overfitting (Figure S3).

Figure 3c shows correct (dark color) and incorrect (light color) predictions for the different models if a threshold value for prediction of LFS or HBC status is arbitrarily placed at a probability value of 0.5. Predictions from leave-one-out cross validation are also shown, where reduced models were produced for all combinations of n-1 variants and then used to predict the disease outcome for the left-out variant (see also Table S5). All 4 models predicted the correct outcome for 16 HBC-variants and 14 LFS-variants. Only 4 variants were incorrectly predicted by all four models, and for these variants the same result was obtained by leave-one-out cross validation. Consistent with the progressive reduction in the C-statistic (Figure 3a), the models make progressively fewer correct predictions as the number of variables in the models was reduced (41, 36, 36 and 35 correct predictions for mod_full, mod_4v, mod_3v and mod_2v, respectively). The reduction in correct predictions is accompanied by a reduced risk of over-fitting (Figure S3) and therefore the reduced models would be expected to perform better than the full model on an independent data set.

Reduced tendency for overfitting was also associated with improved calibration of the reduced models, such that calibration of the mod_2v and mod_3v models was much better than for mod_full and mod_4v (Figure S4). The beta coefficients, *p*-values and odds rations for the reduced models are shown in Table 2. Only two variables in each of the three reduced variable models reach statistical significance, namely the Buried status of variant residues and the predicted difference in their compactness characteristics. In choosing between the models, mod_2v and mod_3v show better calibration compared to mod_4v and mod_full. Since mod_3v gave a slightly higher C-statistic (0.81) compared to mod_2v (0.78) it was selected as the most favored model.

### 3.4. Potential for the Most Favored Model

**Figure 4:**
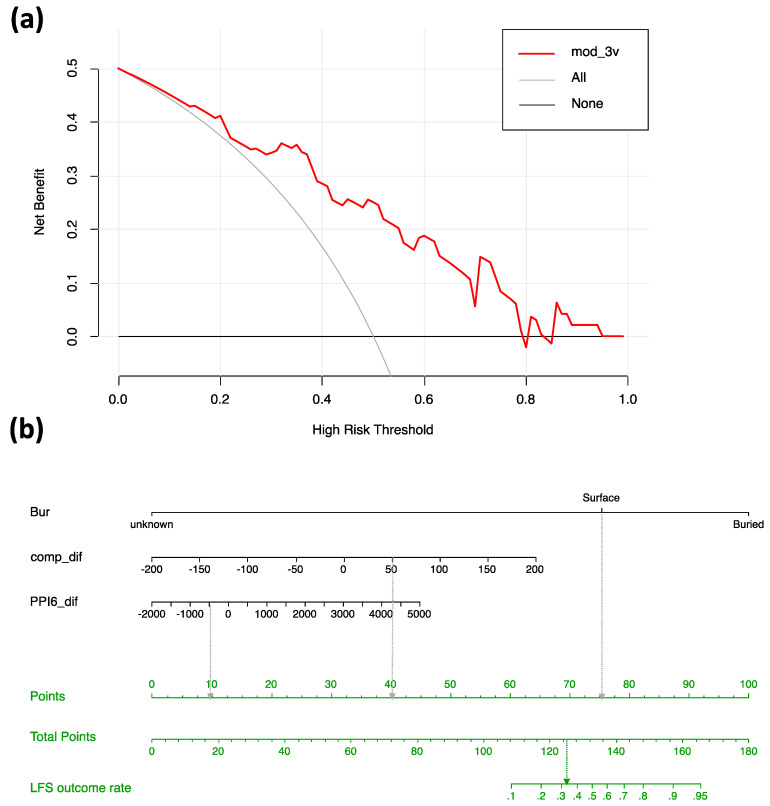
Potential for the most favored model. (**a**) Decision curve analysis of models for prediction of phenotypic outcome (LFS or HBC). The *y*-axis indicates the net benefit of using mod_3v model (red line). The thin gray line (All) shows net benefit values expected assuming all assessed variants are LFS. The darker gray line (None) shows net benefit values expected assuming no assessed variants are LFS. The net benefit for prediction of LFS variants is regarded as positive for probability values exceeding those for the “All” and “None” values. (**b**) The nomogram that facilitates manual estimation of the risk of LFS disease outcome using the mod_3v model. For the value of each explanatory variable the equivalent value on the “Points” scale is assessed. The sum of all Points values is then located on the “Total Points” scale (middle green row) so that the corresponding probability value can be read from the “LFS outcome rate” scale (upper green row). The dotted arrows show a hypothetical example for a surface residue with a comp_dif value of 50 and a PPI6_dif value of −500, for which the equivalent “Point” values (10, 40 and 75) summate to 125 (“Total Points”), giving a LFS outcome risk of slightly over 0.3.

To estimate the potential value of the most favored model (mod_3v) for use in developing improved tools for clinical decision making we used decision curve analysis. The net benefit of using the mod_3v to predict LFS disease outcome at different risk thresholds is shown in Figure 4a. The decision curve for mod_3v provides a higher net benefit than assuming that all potential patients will develop LFS (grey line) at a risk threshold of about 0.2 and out-performs the assumption that no patients will develop LFS (black line) up to a risk threshold of about 0.8. Thus, under conditions where relative LFS prevalence is not extremely low or high, the model would be expected to provide a net benefit if used in the clinical decision-making process. 

Figure 4b shows the mod_3v model in the form of a nomogram that visualizes the prediction model with the respect to the relative importance of the included explanatory variables as well as the way they contribute to a prediction of risk for the alternative disease outcomes. Most important is the classification of Buried or Surface status for the variant residue in the tertiary structure of the TP53 tetramer bound to DNA but the predicted effect of variants on the compactness of the TP53 conformation is also important, with increased compactness of variants increasing the risk for LFS. Changed predicted propensity for protein interaction plays a lesser role with increased interaction propensity of variants increasing the risk for LFS. The values of the Bur variable are already known for all residues in the tertiary TP53 structure used here and it would of course be possible to determine compactness effects (comp_dif) and protein interaction propensity effects (PPI6_dif) values for all possible substitutions of all TP53 residues. Thus, it would be possible to calculate disease outcome risk probabilities for all possible substitutions of residues in the DNA-binding and oligomerization domains if, after due external validation and model development, models similar to those described here were judged to be useful in a clinical setting.

## 4. Discussion

Pathogenic germline variants in *TP53* have classically been associated with Li-Fraumeni syndrome (LFS), a tumor predisposition syndrome with high risk of various childhood as well as adult onset tumors. Increased genetic testing has however revealed that germline *TP53* variants are associated with a broader range of phenotypes, from classical LFS to hereditary breast cancer (HBC), and the outcome may be dependent on both variant characteristics and modifier gene variants elsewhere in the genome [40]. Differential expression of TP53 isoforms has also been discussed to have an impact on cancer risk profile [41,42], but this has mainly been studied in sporadic cancers [43]. The wide variation in the phenotypic outcome in families carrying *TP53* variants creates challenges for the genetic counseling and clinical handling of these individuals.

In an attempt to better understand the molecular basis for the differential disease outcomes associated with different variants and to develop a prediction tool, we studied the impact of germline *TP53* missense variants on protein conformation and their association to disease phenotype. We present a quantitative model that predicts disease outcome (LFS or HBC) as a function of localization of variant residues in the tertiary structure of the TP53 DBD and oligomerization domain together with predicted variant-associated effects on conformation of the full-length protein. 

Our results demonstrate that LFS-variants were enriched in Buried regions (*p* = 0.0014) of the tertiary structure of one or more TP53 monomers in the DNA-bound tetramer, indicating that the set of variant LFS-residues may hypothetically have a larger impact on the folding and overall conformation of the TP53 protein than the set of variant HBC-residues. While the Buried/Surface variable relates to the predisposition of affected residues to lead to LFS or HBC, the compactness (comp_dif) variable is related to how the substitution of the variant residues is predicted to affect compactness, with enhanced compactness favoring the LFS outcome. The protein interaction propensity variable (PPI6_dif) is also positively correlated with the probability of LFS outcome suggesting the importance of protein interactions for the LFS phenotype. These protein interactions could in principle be interactions between monomers within the TP53 tetramer or interactions between the TP53 tetramer and other proteins. Comparison with the positions of residues forming intra-tetramer interactions shows that the major DBD regions predicted to have protein interaction propensity (Figure 1d) contain interface residues between monomers within the TP53 tetramer, suggesting that the PPI6_dif variable may be a measure of effects of variants on the tetrameric integrity of TP53. We cannot of course exclude a role of these regions in other protein interactions. Thus, our favored quantitative model (mod_3v) incorporated three variables encompassing overall topological effects, protein chain related effects and residue-level effects, and it performed acceptably well with a C-statistic of 0.81 as well as having acceptable calibration characteristics and strongly reduced risk of overfitting compared to more complex models. 

Extrapolation of the modelling results suggests that variants that tend to strengthen the tertiary and quaternary structure of the TP53 tetramer would tend to favor the LFS disease outcome. This may be related to the dominant-negative phenotype associated with *TP53* variants that are particularly strongly associated with the LFS phenotype (see Introduction). It could be speculated that a variant which stabilized structural aspects of TP53 monomers and their propensity for tetrameric interactions in relation to wild-type would facilitate the formation of hetero-tetrameric TP53 tetramers in heterozygous individuals, thereby resulting in the dominant-negative phenotype that is observed for many missense variants that are associated with LFS [15].

The HBC-residues are not significantly associated with Buried or Surface status in the structure of wild-type TP53. For the compactness and protein interaction propensity variables, the risk for HBC shows the opposite trend to LFS, since the HBC risk is increased by a decrease in predicted compactness and protein interaction propensity in the mutant proteins. A reasonable speculation would be that the TP53 proteins encoded by HBC-variants are still functional but that the variants cause subtle qualitative or quantitative functional changes that alter the transcriptional output in a way that predisposes carriers to breast cancer but not to other LFS phenotypes. Other explanations are also possible. For example, we cannot exclude that the HBC variants are linked to modifier loci that cause the HBC phenotype and that the HBC outcome is not linked to effects of the *TP53* variants at all. Since the number of patients and families displaying some HBC variants is limited, it is also possible that some variants may subsequently be coupled to LFS. The V157I variant, for example, that is reported in 7 individuals in 2 families was classified as HBC by both IARC and Fortuno et al. (Table S1) although one case with sarcoma was reported in addition to all breast cancer cases in these families. We have not been able to obtain more pedigree information to verify if the family fulfills the LFS-criteria and thereby misclassified. Notably, this variant was by our prediction model predicted as 0.71 likely hood to belong to the LFS-group. While evaluating this model one must be aware of that there is a greater risk that variants within the HBC cohort are misclassified than within the LFS cohort.

None of the included variants affect the main residues involved in interactions with the DNA backbone but several coincide with base-interacting residues as well as with residues important for stabilizing the TP53 tertiary structure. For example, the C277R and R280T variants that are clearly HBC-associated affect residues that make specific interactions with bases in the TP53 binding site and would be likely to affect qualitative or quantitative aspects of DNA binding. Similarly, R249K, V143M and V157I are variants characterized by conservative amino acid substitutions, which are also associated with HBC and affect residues important for the stability of the TP53 tertiary structure. Conceivably, these variants could cause qualitative or quantitative changes to the function of TP53 without having a major negative impact on function. The R282G variant is associated with LFS and affects a residue important for stabilizing the DNA-binding surface in relation to the rest of the TP53 tertiary structure. It could be speculated that this variant disrupts DNA binding activity, which would be likely to cause a dominant-negative phenotype if TP53 hetero-tetramers formed in heterozygous patients.

A limitation of our prediction model is the small cohort of only 24 unique missense variants in each group. The challenge has been to identify cohorts of families with exclusively HBC, especially in the case of *de novo* alterations in breast cancer patients that thus lack information of family history. However, we made an effort to select as clean groups of LFS and HBC variants as possible by following a strict selection procedure (Figure S1) but with the consequence of a limited cohort size. Therefore, there is a need to further evaluate the model in an independent cohort, and if possible with more reliable pedigree information concerning tumor panorama and age of onset, before it is used as a tool for clinical counseling and clinical management, perhaps in combination with other modelling approaches [44]. 

Amadou et al. [17] tried to stratify clinical management according to dominant negative variants and loss of function variants. As families with loss of function variants tend to develop tumors later, they suggested it may be considered to test and screen adults instead of children in those families for the consideration of psychological and financial burdens. Nichols et al. [45] discussed that there were however many cases having the same tumor onset age in families with dominant negative variants as in those with loss of function variants. Therefore, this distinction of *TP53* variants can apparently not be used as a sole guidance for further clinical handling. Instead, we made an attempt to provide a tool for improving genetic counselling and clinical management of these patients and families by creating a prediction nomogram based on the protein conformational impact of the germline missense *TP53* variants. The prediction nomogram (Figure 4b) may support psychological issues in genetic counselling especially in families were the model predicts HBC rather than LFS. However, this model cannot yet be used to stratify for example surveillance programs, as it requires validation in an independent cohort.

## 5. Conclusions

This study explored the relationship between germline *TP53* missense variants and their phenotypic impact, with regard to LFS and HBC, based on a quantitative model combining conformational characteristics of the TP53 protein. Logistic regression models show a clear relationship between disease outcome (LFS or HBC) for *TP53* variants with their effects on aspects of protein conformation and function. The models also appear to have a predictive capacity that may be of practical future use in genetic counselling and management of missense variant carriers. However, there is a need to evaluate the prediction model in an independent cohort prior to any implementation in clinical practice. 

## Figures and Tables

**Table 1:** Explanatory variables related to protein conformation.

Protein Characteristics	Variables	Predictor Algorithm [Ref]
**Tertiary structure propensity**		
buried/surface	Bur	Pymol-findSurfaceResidues script [28]
**Intrinsic protein disorder propensity**		
disorder (trained on Disprot DB)	disprot_dif	Espritz [35]
disorder (trained on NMR structures)	nmr_dif	Espritz [35]
disorder (trained on X-ray structures)	xray_dif	Espritz [35]
disorder (longer regions)	iupl_dif	IUPred2A [36]
disorder (short regions)	iups_dif	IUPred2A [36]
**Predicted protein backbone flexibility**		
protein backbone dynamics	dyn_dif	Dynamine [37]
**Secondary structure propensity**		
alpha-helix/beta-sheet	Sec_dif	Meta-structure [33,34]
**Compactness propensity**		
protein compactness	comp_dif	Meta-structure [33,34]
protein globularity	iupstr_dif	IUPred2A [36]
**Protein interaction propensity**		
protein protein interaction	PPI6_dif	Meta-structure-PPI [33,34]
protein protein interaction	anc_dif	IUPred2A [36]

**Table 2:** Reduced models for multivariate logistic regression analysis of disease outcome.

Intercept and Variable	β	OR (95% CI)	*p-*Value
**Four variables model (mod_4v)**			
Intercept	1.282		0.017 *
Bur			
Surface vs. Buried	−2.465	0.09 (0.02–0.44)	0.003 *
unknown vs. Buried	−9.897	5.03 × 10^−5^(4.31 × 10^−27^–5.87× 10^17^)	0.703
comp_dif	0.023	3.88 (1.30–11.59)	0.015 *
PPI6_dif	0.001	1.29 (0.99–1.67)	0.059
nmr_dif	10.461	2.20 (0.79–6.11)	0.132
**Three variables model (mod_3v)**			
Intercept	1.094		0.030 *
Bur			
Surface vs. Buried	−2.229	0.11 (0.02–0.50)	0.005 *
unknown vs. Buried	−9.03	0.00012 (1.18 × 10^−26^–1.22 × 10^18^)	0.727
comp_dif	0.015	2.40 (1.08–5.30)	0.031 *
PPI6_dif	0.0006	1.15 (0.95–1.39)	0.165
**Two variables models (mod_2v)**			
Intercept	0.914		0.048 *
Bur			
Surface vs. Buried	−1.886	0.15 (0.04–0.62)	0.009 *
unknown vs. Buried	−8.804	0.0002 (1.48 × 10^−26^–1.53 × 10^18^)	0.733
comp_dif	0.014	2.30 (1.04–5.09)	0.039 *

β = beta coefficient; OR = odds ratio; CI = confidence interval; Bur = categorical variable describing whether residues are burried, surface or of unknown location in the TP53 tertiary structure; comp_dif = a continuous variable showing the effect of each variant on the predicted compactness of TP53 at the location of each variant residue (variant value minus wild type value); PPI6_dif and nmr_dif are continuous variables calculated as for comp_dif but reflecting the effect of variant residues on the predicted protein interaction protensity and the predicted intrinsic disorder of TP53, respectively; * = *p* < 0.05.

## Data Availability

Data available in article Supplementary Files.

## Supplementary Materials

The Supplementary Materials are available online at https://www.mdpi.com/article/10.3390/ijms22126345/s1.

## References

[1] Li F.P. (1969). Soft-Tissue Sarcomas, Breast Cancer, and Other Neoplasms. A Familial Syndrome?. Ann. Intern. Med..

[2] Malkin D., Jolly K.W., Barbier N., Look A.T., Friend S.H., Gebhardt M.C., Andersen T.I., Børresen A.-L., Li F.P., Garber J. (1992). Germline Mutations of the p53 Tumor-Suppressor Gene in Children and Young Adults with Second Malignant Neoplasms. N. Engl. J. Med..

[3] Birch J.M., Hartley A.L., Tricker K.J., Prosser J., Condie A., Kelsey A.M., Harris M., Jones P.H., Binchy A., Crowther D. (1994). Prevalence and diversity of constitutional mutations in the p53 gene among 21 Li-Fraumeni families. Cancer Res..

[4] Kleihues P., Schäuble B., Hausen A.Z., Estève J., Ohgaki H. (1997). Tumors associated with p53 germline mutations: A synopsis of 91 families. Am. J. Pathol..

[5] Chompret A., Abel A., Stoppa-Lyonnet D., Brugières L., Pagès S., Feunteun J., Bonaïti-Pellié C. (2001). Sensitivity and predictive value of criteria for p53germline mutation screening. J. Med. Genet..

[6] Martin A.-M., Kanetsky A.P., Amirimani B., Colligon A.T., Athanasiadis G., Shih A.H., Gerrero M.R., Calzone K., Rebbeck T.R., Weber B.L. (2003). Germline TP53 mutations in breast cancer families with multiple primary cancers: Is TP53 a modifier of BRCA1?. J. Med. Genet..

[7] Gonzalez K.D., Noltner K.A., Buzin C.H., Gu D., Wen-Fong C.Y., Nguyen V.Q., Han J.H., Lowstuter K., Longmate J., Sommer S.S. (2009). Beyond Li Fraumeni Syndrome: Clinical Characteristics of Families With p53 Germline Mutations. J. Clin. Oncol..

[8] Frebourg T., Genturis T.E.R.N., Lagercrantz S.B., Oliveira C., Magenheim R., Evans D.G. (2020). Guidelines for the Li–Fraumeni and heritable TP53-related cancer syndromes. Eur. J. Hum. Genet..

[9] Villani A., Shore A., Wasserman J., Stephens D., Kim R.H., Druker H., Gallinger B., Naumer A., Kohlmann W., Novokmet A. (2016). Biochemical and imaging surveillance in germline TP53 mutation carriers with Li-Fraumeni syndrome: 11 year follow-up of a prospective observational study. Lancet Oncol..

[10] Levine A.J. (1997). p53, the Cellular Gatekeeper for Growth and Division. Cell.

[11] Petros A.M., Gunasekera A., Xu N., Olejniczak E.T., Fesik S.W. (2004). Defining the p53 DNA-binding domain/Bcl-xL-binding interface using NMR. FEBS Lett..

[12] Joerger A.C., Fersht A.R. (2008). Structural Biology of the Tumor Suppressor p53. Annu. Rev. Biochem..

[13] Uversky V.N. (2016). p53 Proteoforms and Intrinsic Disorder: An Illustration of the Protein Structure–Function Continuum Concept. Int. J. Mol. Sci..

[14] Wasserman J.D., Novokmet A., Eichler-Jonsson C., Ribeiro R.C., Rodriguez-Galindo C., Zambetti G.P., Malkin D. (2015). Prevalence and Functional Consequence of TP53 Mutations in Pediatric Adrenocortical Carcinoma: A Children’s Oncology Group Study. J. Clin. Oncol..

[15] Freed-Pastor W., Prives C. (2012). Mutant p53: One name, many proteins. Genes Dev..

[16] Bougeard G., Renaux-Petel M., Flaman J.-M., Charbonnier C., Fermey P., Belotti M., Gauthier-Villars M., Stoppa-Lyonnet D., Consolino E., Brugières L. (2015). Revisiting Li-Fraumeni Syndrome from TP53 Mutation Carriers. J. Clin. Oncol..

[17] Amadou A., Achatz M.I., Hainaut P. (2018). Revisiting tumor patterns and penetrance in germline TP53 mutation carriers: Temporal phases of Li–Fraumeni syndrome. Curr. Opin. Oncol..

[18] Cho Y., Gorina S., Jeffrey P., Pavletich N. (1994). Crystal structure of a p53 tumor suppressor-DNA complex: Understanding tumorigenic mutations. Science.

[19] Bullock A.N., Henckel J., Fersht A.R. (2000). Quantitative analysis of residual folding and DNA binding in mutant p53 core domain: Definition of mutant states for rescue in cancer therapy. Oncogene.

[20] Jurneczko E., Cruickshank F.L., Porrini M., Clarke D.J., Campuzano I.D.G., Morris M., Nikolova P.V., Barran P.E. (2013). Probing the Conformational Diversity of Cancer-Associated Mutations in p53 with Ion-Mobility Mass Spectrometry. Angew. Chem. Int. Ed..

[21] Bykov V.J.N., Eriksson S.E., Bianchi J., Wiman K. (2018). Targeting mutant p53 for efficient cancer therapy. Nat. Rev. Cancer.

[22] Olivier M., Goldgar E.D., Sodha N., Ohgaki H., Kleihues P., Hainaut P., Eeles A.R. (2003). Li-Fraumeni and related syndromes: Correlation between tumor type, family structure, and TP53 genotype. Cancer Res..

[23] Li F.P., Fraumeni J.F., Mulvihill J.J., Blattner A.W., Dreyfus M.G., Tucker A.M., Miller R.W. (1988). A cancer family syndrome in twenty-four kindreds. Cancer Res..

[24] Fortuno C., James P.A., Spurdle A.B. (2018). Current review ofTP53pathogenic germline variants in breast cancer patients outside Li-Fraumeni syndrome. Hum. Mutat..

[25] Kharaziha P., Ceder S., Axell O., Krall M., Fotouhi O., Böhm S., Lain S., Borg Å., Larsson C., Wiman K.G. (2019). Functional characterization of novel germline TP53 variants in Swedish families. Clin. Genet..

[26] Bouaoun L., Sonkin D., Ardin M., Hollstein M., Byrnes G., Zavadil J., Olivier M. (2016). TP53Variations in Human Cancers: New Lessons from the IARC TP53 Database and Genomics Data. Hum. Mutat..

[27] Emamzadah S., Tropia L., Halazonetis T.D. (2011). Crystal Structure of a Multidomain Human p53 Tetramer Bound to the Natural CDKN1A (p21) p53-Response Element. Mol. Cancer Res..

[28] Petty T.J., Emamzadah S., Costantino L., Petkova I.D., Stavridi E.S., Saven J.G., Vauthey E., Halazonetis T.D. (2011). An induced fit mechanism regulates p53 DNA binding kinetics to confer sequence specificity. EMBO J..

[29] Script Library PyMOLWiki. https://pymolwiki.org/index.php/Category:Script_Library.

[30] Vertrees J. FindSurfaceResidues—PyMOLWiki. https://pymolwiki.org/index.php/FindSurfaceResidues.

[31] Vertrees J. InterfaceResidues—PyMOLWiki. https://pymolwiki.org/index.php/InterfaceResidues.

[32] Torrance G. Ss–PyMOLWiki. https://pymolwiki.org/index.php/Ss.

[33] Konrat R. (2009). The protein meta-structure: A novel concept for chemical and molecular biology. Cell. Mol. Life Sci..

[34] Mayer C., Slater L., Erat M.C., Konrat R., Vakonakis I. (2012). Structural Analysis of the Plasmodium falciparum Erythrocyte Membrane Protein 1 (PfEMP1) Intracellular Domain Reveals a Conserved Interaction Epitope. J. Biol. Chem..

[35] Walsh I., Martin A., Di Domenico T., Tosatto S.C.E. (2011). ESpritz: Accurate and fast prediction of protein disorder. Bioinformatics.

[36] Meszaros B., Erdos G., Dosztanyi Z. (2018). IUPred2A: Context-Dependent Prediction of Protein Disorder as a Function of Redox State and Protein Binding. Nucleic Acids Res..

[37] Cilia E., Pancsa R., Tompa P., Lenaerts T., Vranken W.F. (2014). The DynaMine webserver: Predicting protein dynamics from sequence. Nucleic Acids Res..

[38] Vickers A.J., Cronin A.M., Elkin E.B., Gonen M. (2008). Extensions to decision curve analysis, a novel method for evaluating diagnostic tests, prediction models and molecular markers. BMC Med. Inform. Decis. Mak..

[39] Liu W., Xie Y., Ma J., Luo X., Nie P., Zuo Z., Lahrmann U., Zhao Q., Zheng Y., Zhao Y. (2015). IBS: An illustrator for the presentation and visualization of biological sequences: Fig. 1. Bioinformatics.

[40] Gargallo P., Yáñez Y., Segura V., Juan A., Torres B., Balaguer J., Oltra S., Castel V., Cañete A. (2020). Li–Fraumeni syndrome heterogeneity. Clin. Transl. Oncol..

[41] Khoury M.P., Bourdon J.-C. (2011). p53 Isoforms: An Intracellular Microprocessor?. Genes Cancer.

[42] Schubert S., De Miranda N., Ruano D., Barge-Schaapveld D., Hes F., Tops C., Joruiz S., Diot A., Bourdon J., Van Wezel T. (2018). PO-059 Cancer-predisposing variants in alternatively spliced TP53 exons. ESMO Open.

[43] Eiholzer R.A., Mehta S., Kazantseva M., Drummond C.J., McKinney C., Young K., Slater D., Morten B.C., Avery-Kiejda K.A., Lasham A. (2020). Intronic *TP53* Polymorphisms Are Associated with Increased *Δ133TP53* Transcript, Immune Infiltration and Cancer Risk. Cancers.

[44] Fortuno C., Cipponi A., Ballinger M.L., Tavtigian S.V., Olivier M., Ruparel V., Haupt Y., Haupt S., Tucker K., International Sarcoma Kindred Study (2019). A quantitative model to predict pathogenicity of missense variants in the TP53 gene. Hum. Mutat..

[45] Nichols K.E., Malkin D. (2015). Genotype Versus Phenotype: The Yin and Yang of Germline TP53 Mutations in Li-Fraumeni Syndrome. J. Clin. Oncol..

