# Supplementary material for: Association between Predicted Effects of TP53 Missense Variants on Protein Conformation and Their Phenotypic Presentation as Li-Fraumeni Syndrome or Hereditary Breast Cancer
Source: Int J Mol Sci. 2021 Jun 14;22(12):6345. doi: 10.3390/ijms22126345 (PMC8231809; doi:10.3390/ijms22126345)
Supplement: Supplementary file 1 [file ijms-22-06345-s001.zip › ijms-1195035-supplementary/Supplementary_figures.pdf]

Figure S1

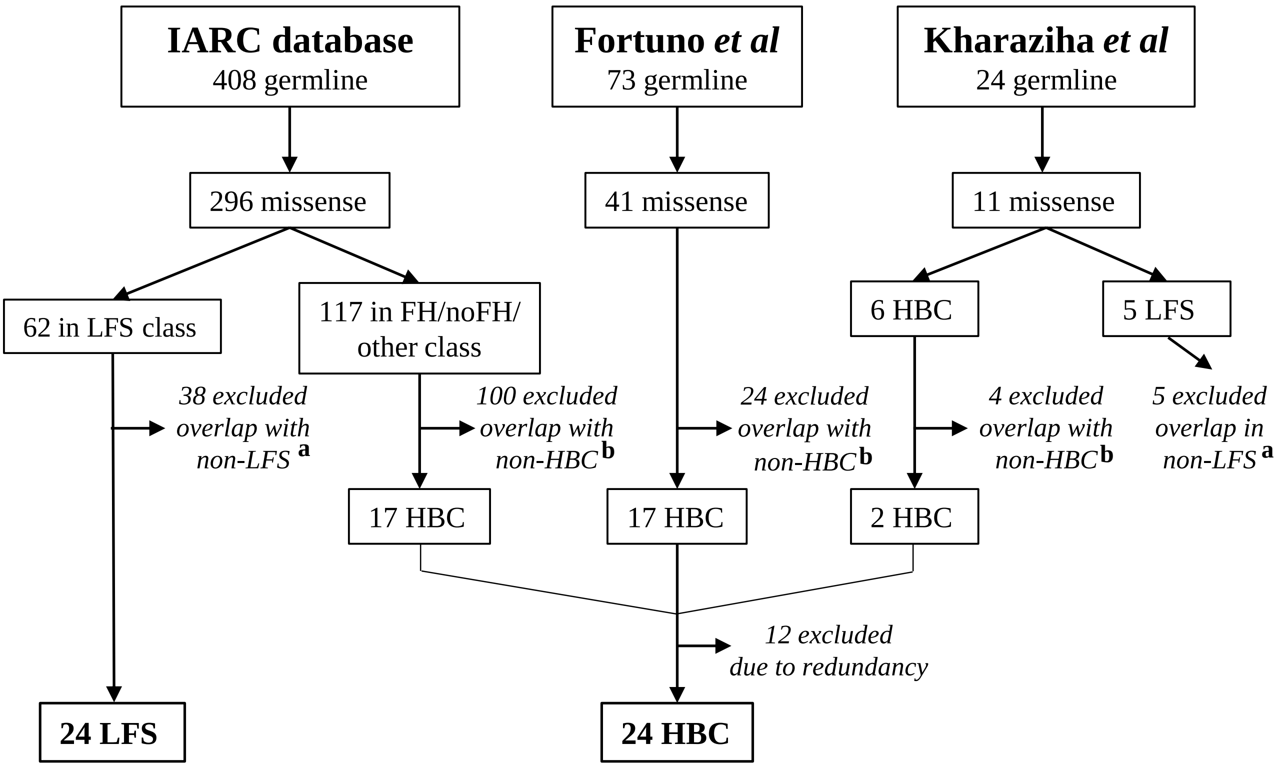

Figure S1. Selection process for the *TP53* missense variants used in the study. The *TP53* missense variants included in the LFS and HBC groups were selected from the IARC database, the review of HBC by Fortuno *et al*[22] and the *TP53* variants identified in the Swedish cohort from 2017, based on Kharaziha *et al*[23].

[a]non-LFS classes refers to: LFL class, TP53 Chompret class, FH class, noFH class and other class in the IARC database.

[b]non-HBC refers to: LFS class, LFL class, TP53 Chompret class and variants without breast cancer history in the IARC database.

Figure S2

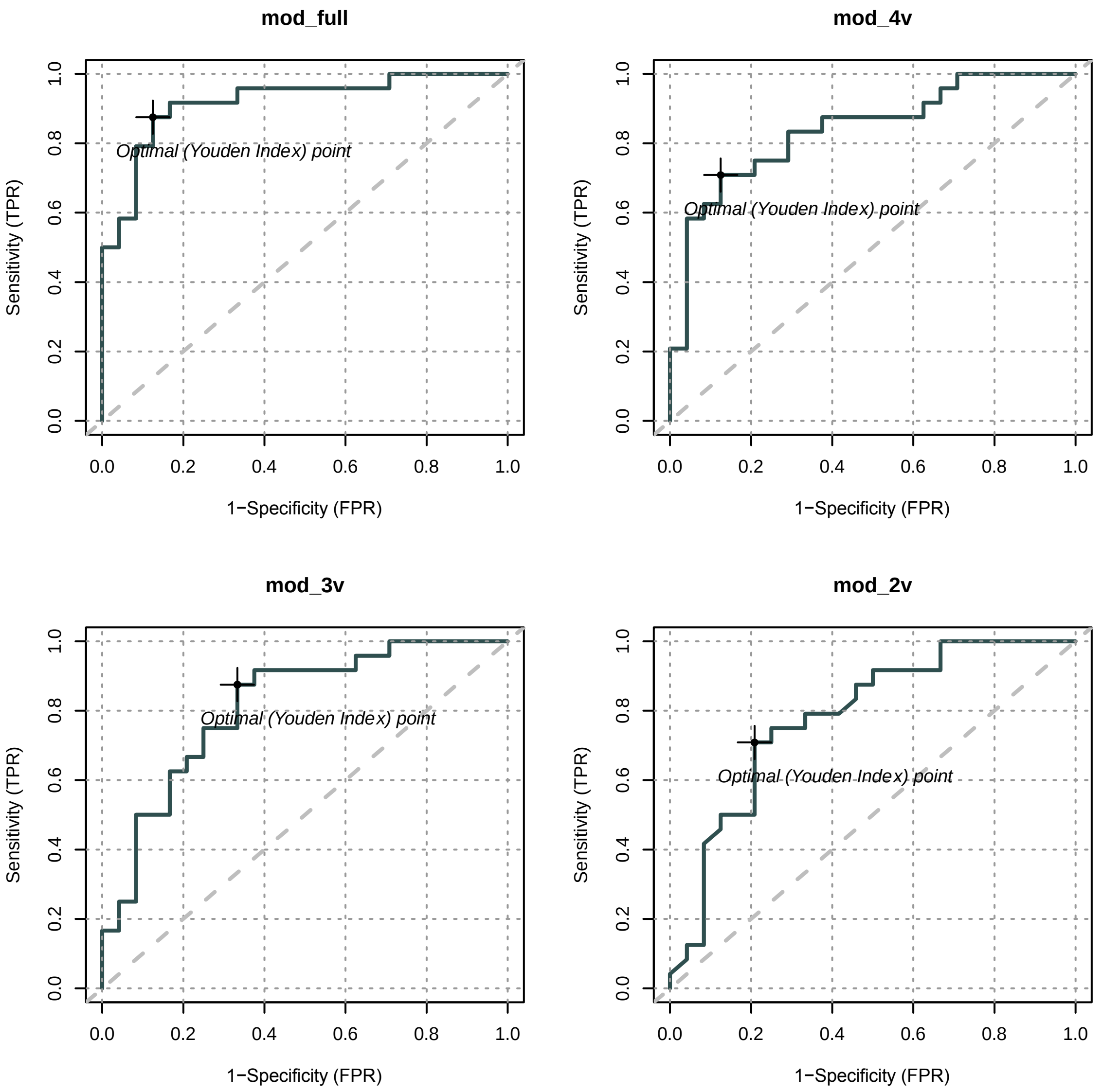

Figure S2. ROC plots for the full model (12 variables) and reduced models with four, three and two explanatory variables. The models are assessed by the relation ship between their true positive rate (TPR, sensitivity) and their false positive rate (FPR, 1-specificity). The Youden index point shows the point at which sensitivity and specificity are optimally maximized. The full model performs somewhat better than the reduced models as reflected in a higher C-statistic (see Figure 3A).

Figure S3

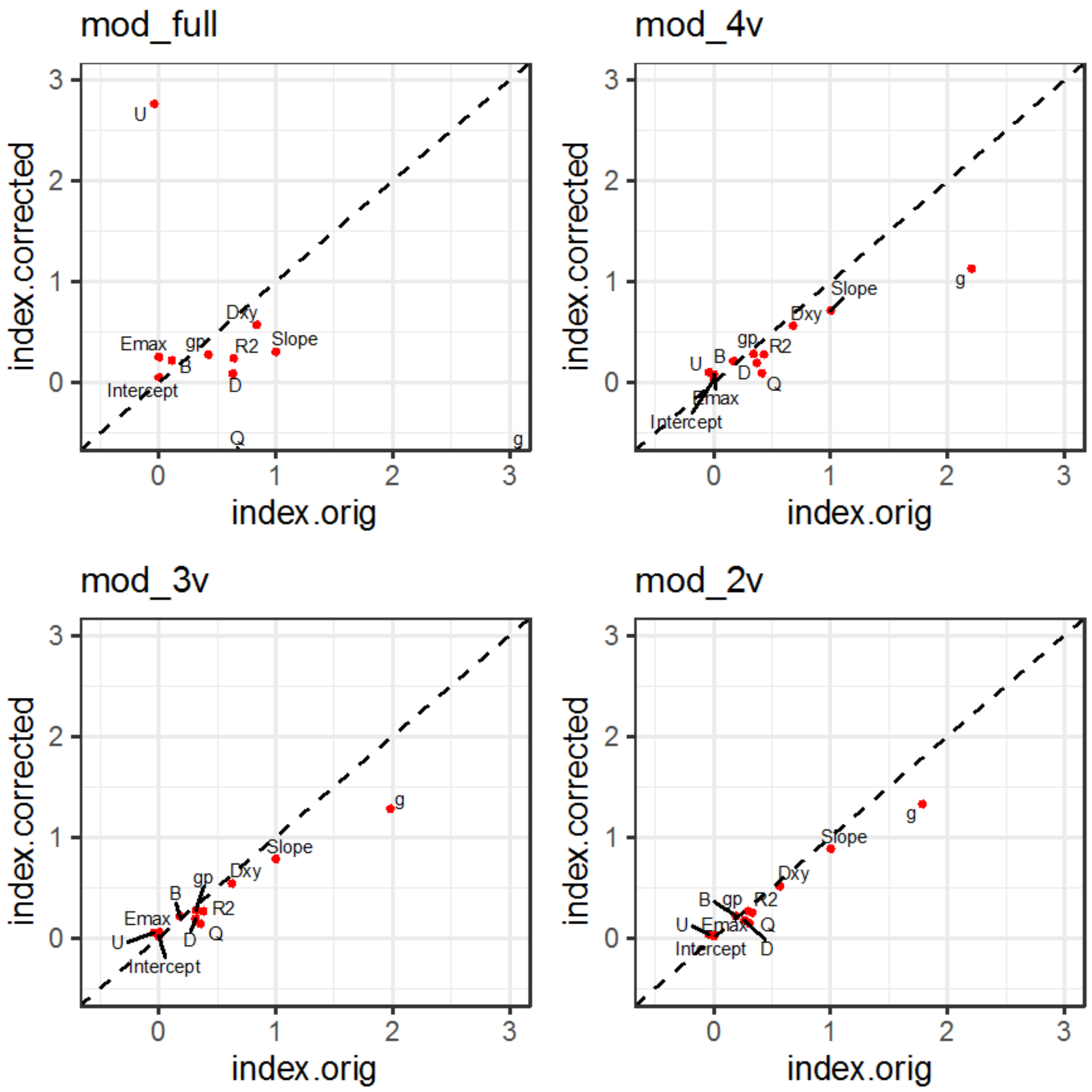

Figure S3. Comparison of model quality criteria for the full model (12 variables) and reduced models with four, three and two explanatory variables. Values after correction for overfitting (index.corrected) are plotted in relation to values before correction (index.orig) in order to compare the extent of estimated overfitting for the different models. In the absence of any estimated overfitting, the plotted values should be equal and thus lie on the dashed line. The quality criteria plotted were calculated by the R rms::validate function (see package documentation for explanation of individual criteria). Note that the values for Q (x = 0.66, y = -2.20) and g (x = 3.35, y = -7.05) are far outside the boundaries of the plot for the full model, which shows much greater signs of overfitting than the reduced models.

Figure S4

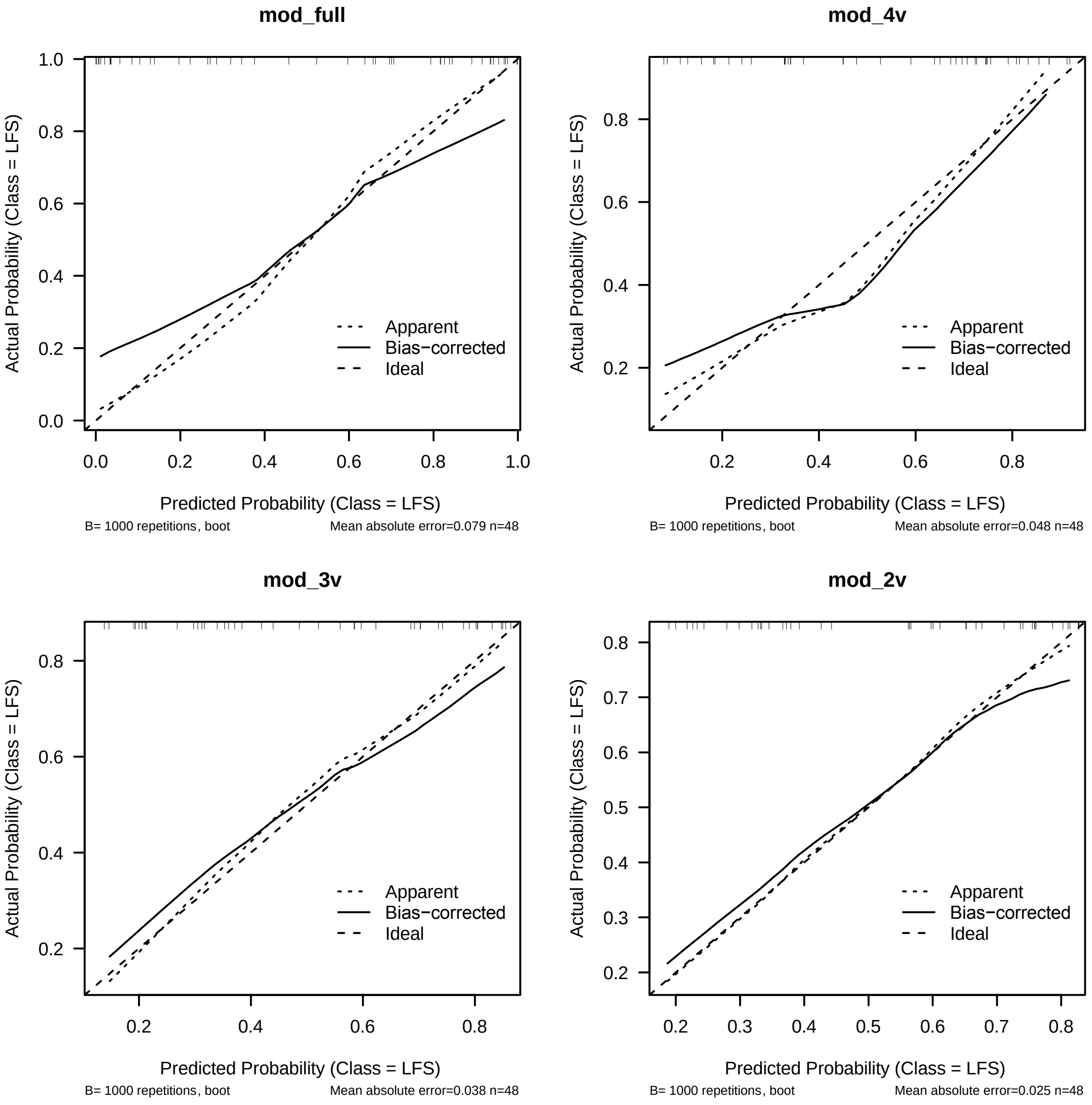

Figure S4. Calibration comparison for the full model (12 variables) and reduced models with four, three and two explanatory variables. The model predicted probabilities of classification as LFS are plotted in relation to the actual probabilities. The Ideal line (dashed) shows the expected plot for perfect models. The Apparent line (dotted) shows the performance of the models and the Bias-corrected line (solid) shows the model performance expected after correction. The comparison shows that variable reduction is associated with better calibrated models.
