# Supplementary material for: Association between Predicted Effects of TP53 Missense Variants on Protein Conformation and Their Phenotypic Presentation as Li-Fraumeni Syndrome or Hereditary Breast Cancer
Source: Int J Mol Sci. 2021 Jun 14;22(12):6345. doi: 10.3390/ijms22126345 (PMC8231809; doi:10.3390/ijms22126345)
Supplement: Supplementary file 1 [file ijms-22-06345-s001.zip › ijms-1195035-supplementary/Supplementary_materials and methods.pdf]

ppSupplementary information to:

Association between predicted effects of *TP53* missense variants on Protein Conformation and their Phenotypic Presentation as Li-Fraumeni Syndrome or Hereditary Breast Cancer

Yaxuan Liu, Olga Axell, Tom van Leeuwen, Robert Konrat, Pedram Kharaziha, Catharina Larsson, Anthony Wright, Svetlana Bajalica Lagercrantz

Materials and Methods

Meta-Structure based Prediction of Protein-Protein Interaction Sites

Meta-structure [33] data was used to predict protein interaction sites in the TP53 sequence using a meta-structure-based homology method to interrogate a set of known protein-protein interaction interfaces as already reported [34]. Secondary structure elements are defined as in the originally described meta-structure method [33]. However, here we use a slightly different procedure to calculate compactness values. The statistical distribution function, (,A,B,l_{AB}), quantifies the topological information (probability of finding a particular shortest path length (value) for two residues of type A and B separated by l_{AB} positions in the primary sequence) [33]. The input primary sequence is used to predict the probability of being a direct contact, i.e. =1, for each possible amino acid pair (of residue type A and B and separated by l_{ab} in the sequence). The residue specific compactness value is then calculated by C_i = N_1* (,A_i,B_j,|i-j|)-N_0, $\forall(i\neq j)$. N_0 and N_1 are empirical scaling factors that ensure

that highly exposed residues (low compactness residues) display C_i values close to 0 [33]. Individual C_i values thus correspond to the probability (sum of individual contact probabilities) of a given residue being surrounded by other residues (overall, the larger the compactness value the more residues neighbours/contacts are observed).

The availability of quantitative meta-structure information on a per-residue basis provides the possibility to perform protein homology searches that go beyond the primary sequence level. This aspect is exploited in the sequence-derived protein-protein interaction site analysis. The computational strategy is based on the rational that there is a (limited) set of protein interaction motifs with distinct meta-structural properties and that structurally similar epitopes will display significant meta-structure homology. The meta-structure similarities can be quantified and used as the basis for the binding site prediction. Thus, the task in the analysis of a given query protein is to identify meta-structurally similar epitopes in a database of known protein-protein complexes (i.e. the PDB structure database). First, as a basis for the calculation about 1750 sequences of protein complexes were first extracted from the PDB database (www.rcsb.org) together with the information identifying residues that are part of the interaction sites. Residues located in interaction sites were selected using the criteria that the C -C distances to the nearest neighbor residues of the binding partner protein are below a cutoff of 8Å. Secondly, the meta-structure parameters (compactness and local secondary structure) were calculated for the selected protein sequences from the selected protein complexes. To predict putative interaction sites for a query protein the strategy is as follows: The sequence of a query protein is compared to all template sequences from the dataset based on meta-structure similarity. Residues of the query protein aligning with template residues that are part

of interaction sites are identified and allocated weights corresponding to the alignment (similarity) scores. The summation over all individual alignments thus provides a reliable estimation of the protein interaction propensity, which is expressed as the protein-protein interaction score.

In order to speed up the calculation and allow for large-scale (high-throughput) applications a strategy reminiscent of the shotgun approach in genome sequencing was employed. Briefly, the combination of residue-specific C_i and L_i values are converted into a (pseudo) one-letter code. To this end, the range of C_i and L_i values are each divided into 5 bins (resulting into a total of 25 combinations). The residue-specific meta-structure information of a query protein is converted into a 1-dimensional string and sequence alignment thus reduced to simply identifying string identity. For computational speed the total string for the query protein is progressively cut into smaller segments of decreasing length and the individual string segments are analyzed. For each and every string segment the template (sequence of the protein complex) string is searched for the existence of an identical string segment. In case of an identical string segment in the template and provided that the mapped residues of the template are part of an interaction site the corresponding residues of the query protein are considered to be part of a protein-protein interaction site as well and will be given a statistical weight proportional to the length of the segment. Summation over all alignments with different segments lengths and all template strings gives the final protein-protein interaction score. In practice, the segment length is varied between 6 (or 10) and 60 residues. String identity is hardly found for larger segment lengths and smaller segment lengths are too abundant (uninformative) and only contribute to statistical noise.

The performance of the protein interaction site predictor was statistically evaluated by comparing predicted interaction sites with available structural information from the PDB protein dataset. In the prediction, of course, sequence similarities between query and template protein were considered and only dissimilar sequences were used in the calculation. The statistical evaluation was as follows: First, the protein interaction scores were calculated and the most probable interaction epitopes were identified based on the score. Only epitopes displaying at least 10% (or 5%) of the maximum interaction score were considered. In the statistical evaluation, we then evaluated how many of the residues in the interaction interface (experimentally identified based on the 3D structure) of the individual protein complexes are correctly predicted by the algorithm. A correct prediction is the case if the interface residue is part of an identified (predicted) epitope. In the template data set the number of interaction site residues (for a given protein) varies between 6 and about 100 residues. We thus decided to base the statistical evaluation on whether epitopes (with varying length) are correctly identified rather than individual residues. For an epitope length of 20 amino acids correct predictions were achieved for 62% (10% cutoff) and 77% (5% cutoff), while for a 30 aa epitope length the performance improved to 72% (10% cutoff) and 86% (5% cutoff).
